# Facile isothermal solid acid catalyzed ionic liquid pretreatments to enhance the combined sugars production from *Arundo donax* Linn.

**DOI:** 10.1186/s13068-016-0589-8

**Published:** 2016-08-24

**Authors:** Tingting You, Lupeng Shao, Ruizhen Wang, Liming Zhang, Feng Xu

**Affiliations:** Beijing Key Laboratory of Lignocellulosic Chemistry, Beijing Forestry University, Beijing, 100083 China

**Keywords:** *Arundo donax* Linn., Acid enhanced ionic liquid pretreatment, Enzymatic hydrolysis, Total reducing sugars

## Abstract

**Background:**

Solid acid catalyzed inexpensive ionic liquid (IL) pretreatment is promising because of its effectiveness at decreasing biomass recalcitrance to subsequent enzymatic hydrolysis or in situ hydrolysis of carbohydrate oligomers. However, the conventional strategy was limited by the complex non-isothermal process and considering only one aspect of sugar recovery. In this study, facile isothermal pretreatments using Amberlyst 35DRY catalyzed 1-*n*-butyl-3-methylimidazolium chloride ([C_4_mim]Cl) at mild conditions were developed on bioenergy crop *Arundo donax* Linn. to enhance the combined sugars released. The physicochemical differences, enzymatic digestibility, and sugars released in situ were evaluated and compared to define the best set of conditions.

**Results:**

The optimized isothermal pretreatment (110 °C, IL for 3 h, Amberlyst for 1 h) resulted in significant enhancement in combined sugars released (58.4 g/100 g raw materials), recovering 85.0 % of the total reducing glycan in the raw biomass. This remarkable improvement could be correlated to cellulose crystallinity reduction, crystalline conversion, and partial removal of the main chemical components caused by the pretreatment. Particularly, solubilization of hemicelluloses and partial depolymerization of cellulose contributed to the synergetic improvement of sugars production in enzymatic hydrolysis and in situ. Irrespective of the generous differences in mass recovery, the highest cellulose digestibility of 90.2 % and sugar released of 43.0 % (based on initial materials) in the pretreatment liquor were obtained. Interestingly, lignin (0.8–6.1 %) and sugars derived lactic acid (4.70–5.94 %) were produced without any notable deleterious effects.

**Conclusions:**

Isothermal [C_4_mim]Cl-Amberlyst pretreatment was a highly effective, simple, and convenient process that produced high yields of fermentable sugars from recalcitrant biomass by in situ hydrolysis of soluble biomass and enhancement of cellulose digestibility of the regenerated biomass. Relatively high amount of new revenues beyond sugars of this pretreatment could promote the commercial viability.

**Electronic supplementary material:**

The online version of this article (doi:10.1186/s13068-016-0589-8) contains supplementary material, which is available to authorized users.

## Background

The world’s ever-growing energy demands and concerns over the shortage of oil reserves accompanying with its detrimental effects on climate change have garnered interest in the research for alternative renewable sources for the sustainable production of fuels and petroleum based products [1]. Dedicated bioenergy crops, such as switchgrass, miscanthus, and reed are renewable and present in great abundance that can be converted to transportation fuels and commodity chemicals [2, 3]. A perennial grass *Arundo donax* Linn. is a promising candidate when compared to other bioenergy crops because of its high biomass production, plant adaptability, and low input required for its cultivation [2]. Studies investigating the conversion of this biomass to fermentable sugars would bring economic and ecological benefits. Due to the naturally rigid and complex submicroscopic structure of plant, pretreatment is a pre-requisite step to modify the physical and chemical properties and to enhance enzymes accessibility to achieve high yields of fermentable sugars [4].

Ionic liquids (ILs) have held great promise as powerful green solvents for pretreatment and dissolution of biomass with improved yields of reducing sugars [5–9]. This process has certain advantages such as environmentally benign, feedstock agnostic, and short processing time demanded to convert pretreated biomass to high yields of fermentable sugars [5, 10–12]. However, the high cost of IL impedes the industrial investment of this technology. Up to now, combinatorial pretreatments involving ILs and other chemicals have been developed to cut down the high cost of ILs whist boosting the sugars production [13–15]. Among them, acid in inexpensive prototype 1-*n*-butyl-3-methylimidazolium chloride ([C_4_mim]Cl) pretreatments have been considered as cost-effective alternatives to the expensive IL to disrupt the physical and biochemical barriers for enzymatic hydrolysis [15]. In the earlier works, in situ hydrolysis of carbohydrate oligomers that dissolved in IL to monomers with acid catalysts was proposed [16, 17]. Nevertheless, there exists a void of explicit knowledge regarding the promotion to the combined sugars production by in situ hydrolysis of IL-soluble biomass and enhancement of cellulose digestibility of regenerated biomass after pretreatment across techniques. For instance, Sun and coworkers [17] have tended to focus only on the production and extraction of sugars produced in IL. The evaluation of the potential sugar production from regenerated solids was ignored after the pretreatment.

Maximizing valorization of lignocellulosic biomass can be achieved by neoteric methods using ILs and acid catalysts [18]. Fractionation of biomass with ionic liquid-acid systems to cellulose-rich fractions is believed to facilitate cellulose conversion [15]. Compared to the liquid acids, solid acids such as nafion, Amberlyst resins, and zeolites are less toxic, easy to recycle, and substantially mitigating the undesired degrading compounds in pretreatment liquors [19]. Moreover, a few compounds of commercial interest, such as phenolic acid and lactic acid can be directly accessible from biomass by using heterogeneous solid acid in ILs [20–22]. For instance, da Costa Lopes and coworkers [21] used acid resin Amberlite XAD-7 to extract phenolic compounds that dissolved after a complete fractionation of biomass by IL. In our previous research, a two-step solid acid catalyzed [C_4_mim]Cl pretreatment has been developed to open the rigid structure of plants [23]. Accordingly, 5.21-fold enhancement of fermentable sugars yield from *A. donax* could be gained by enzymatic hydrolysis. More recently, new acidic ILs have been therefore designed to efficiently separate cellulose-rich fractions from lignocellulosic biomass to enhance the cellulose digestibility [6, 7, 9].

However, there are still some limitations inherent for the practical application of IL-solid acid pretreatments despite satisfactory sugars yields have been obtained. The previous proposed procedures [15, 23] required a comparatively high temperature (160 °C) in the first IL pretreated step, which was higher than the maximum operating temperatures of most resins. Therefore, additional cooling apparatus was demanded to cool down the system before adding any acid resins. The extra procedure makes the pretreatment more complex and poses pivotal equipment cost and energy challenge when considering as part of a practical, large-scale biomass pretreatment process. In addition, chloride based ILs were reported to show signs of decomposition at temperatures of 160 °C [24]. The high temperature used in the state-of-the-art methods might deteriorate ILs quality for reuse. Furthermore, the in situ sugars production directly during the pretreatment was out of consideration when evaluated the effectiveness of pretreatments. To advance the pretreatment technology, we describe a facile isothermal pretreatment that used Amberlyst 35DRY catalyzed [C_4_mim]Cl to achieve high combined fermentable sugars yields from bioenergy crops *A. donax* at mild conditions. The effects of pretreatments on the chemical composition, cellulose digestibility, structural features, and reducing sugars released in pretreatment liquors were detected and compared to find out the optimal condition. Other value-added chemicals were observed as the new revenues beyond sugars. The in-depth knowledge of this approach will be beneficial for raising the profitability of the entire biorefinery.

## Results and discussion

### Biomass isothermal pretreatment and dry mass recovery

A facile isothermal protic acid resin catalyzed ionic liquid pretreatment was utilized to deconstruct *A. donax* cell wall. To define the best set of conditions for pretreatment, *A. donax* was processed using different pretreatment combination of temperature and acid reaction time and the yields of recovered samples are compared in Table 1.

**Table 1:** Mass recovery and crystallinity of the raw and pretreated *A. donax* under different conditions

Samples	Pretreatment conditions		Recovered *A. donax* (%)	CrI (%)	LOI
	[C_4_mim]Cl	Amberlyst 35DRY			
Raw	Untreated	Untreated	100	36.0	1.43
R90-1.0	90 °C 3 h	90 °C 1.0 h	95.4	29.4	0.93
R100-1.0	100 °C 3 h	100 °C 1.0 h	78.4	31.4	0.58
R110-1.0	110 °C 3 h	110 °C 1.0 h	58.0	24.7	0.63
R120-0.5	120 °C 3 h	120 °C 0.5 h	66.1	23.2	0.53
R120-1.0	120 °C 3 h	120 °C 1.0 h	46.7	25.8	1.03
R120-1.5	120 °C 3 h	120 °C 1.5 h	31.7	27.3	0.56
R130-0.5	130 °C 3 h	130 °C 0.5 h	53.5	25.2	0.86
R140-0.5	140 °C 3 h	140 °C 0.5 h	28.2	25.8	1.15
R150-0.5	150 °C 3 h	150 °C 0.5 h	25.8	16.5	13.78

Temperatures of 90–150 °C and acid reaction time intervals of 0.5–1.5 h (Table 1) were used to avoid the degradation of Amberlyst 35DRY while reducing biomass recalcitrance. As expected, the dry mass recovery decreased gradually with increasing temperature and prolonging acid reaction time, ranging from 95.4 % for the R90-1 sample to 25.8 % for the R150-0.5 sample. After pretreatment at 90 °C and Amberlyst catalyzed for 1.0 h (R90-1.0), only 4.6 % biomass was consumed by the system, but more than 70 % biomass was lost after pretreatment at 150 °C and acid catalyzed for 0.5 h (R150-0.5). It is probably the consequences of severe degradation of carbohydrates and partial removal of lignin by IL-acid at high temperatures. Moreover, the *A. donax* pretreated at high temperatures likely reacted with the ionic liquid and formed a highly viscous paste that could increase the mass loss. It is interesting to note that the mass loss of R130-0.5 sample is lower than that of R120-1.0 and R120-1.5 samples. These values differed from those described in the literature by Trinh et al. [12], which indicated that the pretreatment of softwood at 130 °C in [C_4_mim]Cl resulted in considerable decrease in the solid yields than that at 120 °C. Rinaldi and coworkers [16, 25] reported that Amberlyst released hydronium ions (H_3_O^+^) in IL progressively to selectively cleave the longer cellulose chain and other soluble compounds to oligomers and sugars. Amberlyst reaction time was therefore suggested to be the main controlling factor at a high incubation temperature for mass recovery from *A. donax*.

### Chemical composition of pretreated *Arundo donax*

Chemical composition determination is of vital importance to understand the biomass conversion. The chemical compositions of raw *A. donax* and of samples submitted to IL-Amberlyst pretreatments are illustrated in Table 2. As can be seen, the raw *A. donax* used in this study presents cellulose (44.59 %), hemicellulose (17.15 %), and lignin (20.54 %) composition similar to those reported in the literature for the same material, whose values vary from 29.2–42.15 % for cellulose, 19.2–32 % for hemicellulose and 19.20–24.3 % for lignin [2, 26]. Significant differences in chemical compositions were observed in the IL-Amberlyst pretreated samples.

**Table 2:** Chemical composition of the raw and pretreated *A. donax* obtained after isothermal [C_4_mim]Cl-Amberlyst 35DRY pretreatments

Samples	Cellulose	Hemicelluloses	AIL^a^	ASL^b^	Ash	Acetyl
Raw	44.59 (44.59)^c^	17.52 (17.52)	19.15 (19.15)	1.39 (1.39)	0.81 (0.81)	4.91 (4.91)
R90-1.0	44.77 (42.71)	17.15 (16.36)	18.19 (17.35)	1.2 (1.14)	1.09 (1.04)	4.70 (4.49)
R100-1.0	52.38 (41.07)	15.7 (12.31)	19.06 (14.94)	1.25 (0.98)	1.11 (0.87)	4.89 (3.84)
R110-1.0	56.56 (32.80)	11.21 (6.50)	23.27 (13.50)	1.32 (0.77)	1.66 (0.96)	4.22 (2.45)
R120-0.5	51.39 (33.97)	14.13 (9.34)	22.73 (15.02)	1.17 (0.77)	1.23 (0.81)	4.24 (2.79)
R120-1.0	47.43 (22.15)	11.65 (5.44)	25.35 (11.84)	1.03 (0.48)	1.72 (0.80)	4.31 (2.01)
R120-1.5	42.99 (13.63)	9.51 (3.01)	34.46 (10.92)	1.06 (0.34)	1.81 (0.57)	3.99 (1.27)
R130-0.5	54.24 (29.02)	13.85 (7.41)	23.72 (12.69)	1.21 (0.65)	1.57 (0.84)	4.64 (2.48)
R140-0.5	46.14 (13.01)	8.6 (2.43)	39.22 (11.06)	1.12 (0.32)	2.6 (0.73)	4.84 (1.36)
R150-0.5	23.62 (6.09)	10.19 (2.63)	46.99 (12.12)	1.03 (0.27)	2.00 (0.52)	4.77 (1.23)

Data refer to the samples, in terms of cellulose, hemicellulose, AIL, ASL, ash, and acetyl contents (w/w, dry weight basis). Data are means of three replicates

^a^acid insoluble lignin

^b^acid soluble lignin

^c^relative content (mass recovery × relative content)

Ionic liquid typically with 1,3-dialkylimidazolium cations can liquefy both lignin and polysaccharides in plant cell wall and then can be reprecipitated from solutions by adding anti-solvents, for examples, water and ethanol [27, 28]. During the selective regeneration, the major cell wall components were partially separated. Table 2 shows that the isothermal [C_4_mim]Cl-Amberlyst pretreatments contributed to the removal of hemicellulose in *A. donax*. The hemicellulose percentage decreased with elevated temperature and prolonged time of acid reaction. R140-0.5 sample displayed the lowest hemicellulose content of 8.60 % (2.43 %) after the isothermal IL-Amberlyst pretreatment at 140 °C and acid catalyzed for 0.5 h. This is primarily because both the acid and ionic liquid pretreatment at a high temperature favor the solubilization and chemical degradation of hemicellulose [29]. In an earlier work, the effect of [C_4_mim]Cl-hydrochloric acid pretreatment on grass samples was investigated (5 % solid loading, at 160 °C for 1.5 h followed by 15 min aicdolysis by 4 M HCl) [17]. They also reported that IL-acid pretreatment had a major effect on hemicellulose and a xylan content of 1.1 % was reported for the solid residue. Moreover, the acetyl contents in the pretreated samples were slightly decreased (Table 2), which may facilitate the enzymatic hydrolysis.

Cellulose was more susceptible to the high pretreatment temperature and long acid reaction time. The isothermal pretreatments resulted in 1.88–38.5 % cellulose removal. Cellulose in the pretreated *A. donax* samples was gradually decreased with increasing pretreatment temperature and acid reaction time, reaching a minimum of 6.09 % at 150 °C, acid catalyzed for 0.5 h. The noticeable degradation of cellulose may ascribe to severe cellulose depolymerization at high temperature in IL-acid system [25]. Irrespective of mass loss, the relative content of cellulose in samples considering the same acid reaction time (1.0 or 0.5 h), was firstly increased, and then decreased as the temperature increased with peak values of 56.56 % at 110 °C, acid catalyzed for 1.0 h and 54.24 % at 130 °C, acid catalyzed for 0.5 h (Table 2). It is noted that the low relative contents of cellulose are found in R90-1.0, R120-1.0, R120-1.5, R140-0.5, and R150-0.5 samples.

Lignin in biomass was partially removed by IL with evidence of decreased lignin amount in the regenerated residues (11.26–18.49 %, including AIL and ASL fractions). The dissolution of hemicellulose into IL-acid system contributed to a more efficient removal of lignin. A similar tendency of lignin removal to hemicellulose solubilization could be detected as lignin was covalently linked to hemicellulose to some extent. However, the relative content of lignin in pretreated samples was only slightly decreased in R90-1.0 and R100-1.0 samples but obviously increased (23.90–48.02 %) in the others. Relative contents of lignin in R90-1.0 and R100-1.0 samples were similar to those reported previously using a two-steps IL (160 °C)-Amberlyst (90 °C) pretreatment [23]. The highest relative content of lignin was observed in R150-0.5 sample, accompanying the lowest cellulose content. We attribute this high lignin content to the comprehensive functions of cellulose hydrolysis, hemicellulose solubilization, lignin redistribution, and the possible formation of insoluble substrates from carbohydrate conversion during acid pretreatment at high temperature [16, 30, 31]. Further examinations are needed to find out the dominating determinant.

### Enzymatic digestibility of pretreated *Arundo donax*

**Fig. 1:**
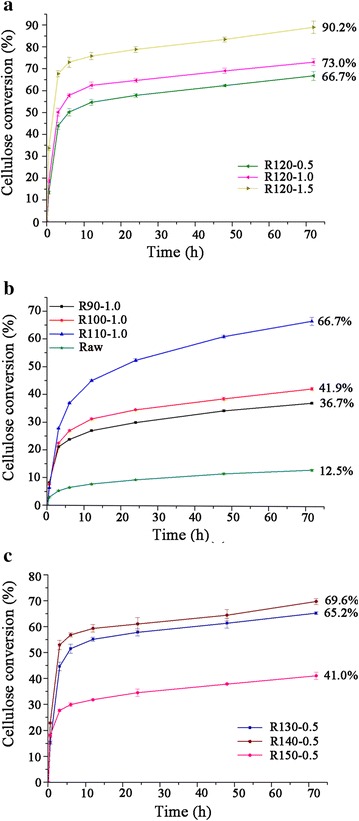
Cellulose conversions of raw and pretreated *A. donax*. by enzymatic saccharification. Cellulose conversions expressed as percentage of the theoretical glucose in pretreated samples. **a** Effect of solid acid reaction time (0.5, 1.0, and 1.5 h) on enzymatic hydrolysis, **b** Effect of low pretreatment temperature (90, 100, and 110 °C) on enzymatic hydrolysis, and **c** effect of high pretreatment temperature (130, 140, and 150 °C) on enzymatic hydrolysis. *Error bars* indicate standard deviation of triplicate determinations. Sample code with definition is in Table 1

Figure 1 presents the temporal profiles of cellulose conversion from raw and pretreated *A. donax* samples under various pretreatment conditions. Since IL in substrate showed toxic or inhibitory effects on microorganisms’ growth, all the samples were washed thoroughly before enzymatic hydrolysis [32]. Enzymatic saccharification assays were conducted under the same conditions to ensure the validity of comparison. As can be seen, *A. donax* substrates originating from IL-Amberlyst pretreatments were readily degraded by enzymes when compared to raw materials-enzymatic digestibilities were significantly enhanced and high cellulose conversion and high hydrolysis rate were observed.

Our results showed evidences that enzymatic saccharification enhancement depends on the Amberlyst reaction time considering the same temperature at 120 °C (Fig. 1a). The digestibilities of samples exposed to water pretreatments of the same temperature profiles were also evaluated as the heat alone might increase the cellulose digestibility. As can be seen from Additional file 1, *A. donax* subjected to IL-Amberlyst pretreatment displayed an increase of 5.32, 5.58, 6.25 folds in cellulose digestibility compared to the corresponding water pretreated samples after 0.5, 1.0, and 1.5 h of Amberlyst pretreatment, respectively. The highest cellulose conversion was obtained for *A. donax* pretreated at 120 °C and acid catalyzed for 1.5 h. After 72 h of enzymatic hydrolysis, the glucose yield was reached up to 90.2 % relative to initial level in the corresponding pretreated biomass (Fig. 1b). This was consistent with previous observations employed [C_4_mim]Cl at a long pretreatment time or dilute acid at high temperature [12, 29]. For example, studies conducted by Trinh and coworkers [12] obtained ~90 % cellulose digestion when the wood materials were pretreated with [C_4_mim]Cl at 120 °C for 24 h. Nevertheless, Uppugundla and coworkers [29] achieved nearly 100 % glucan conversion for corn stover pretreated with 1-ethyl-3-methylimidazolium acetate ([C_2_mim][OAc]) for 3 h at 140 °C. As the cost of IL and pretreatment temperature are very important parameters for an economic analysis of IL pretreatment, the comparatively low price of [C_4_mim]Cl (approximately 1/60th of [C_2_mim][OAc]) and depressed temperature used in the present study may compensate the glucose release [18, 33].

Samples pretreated at higher temperature with shorter acid reaction time or lower temperature with longer acid reaction time showed similar enhanced cellulose digestibilities [34]. It is interesting to note that the cellulose digestibility increases dramatically with temperature increases from 90 to 110 °C, whereas decreased increments are detected when the temperature increases from 130 to 150 °C (Fig. 1b, c). According to literature, the presence of lignin in biomass hinders the enzymatic hydrolysis by nonproductive binding of cellulase to its surface [35]. High relative content of lignin in R150-0.5 sample (Table 2) could be the major factor that restricted the augment of cellulose conversion.

**Fig. 2:**
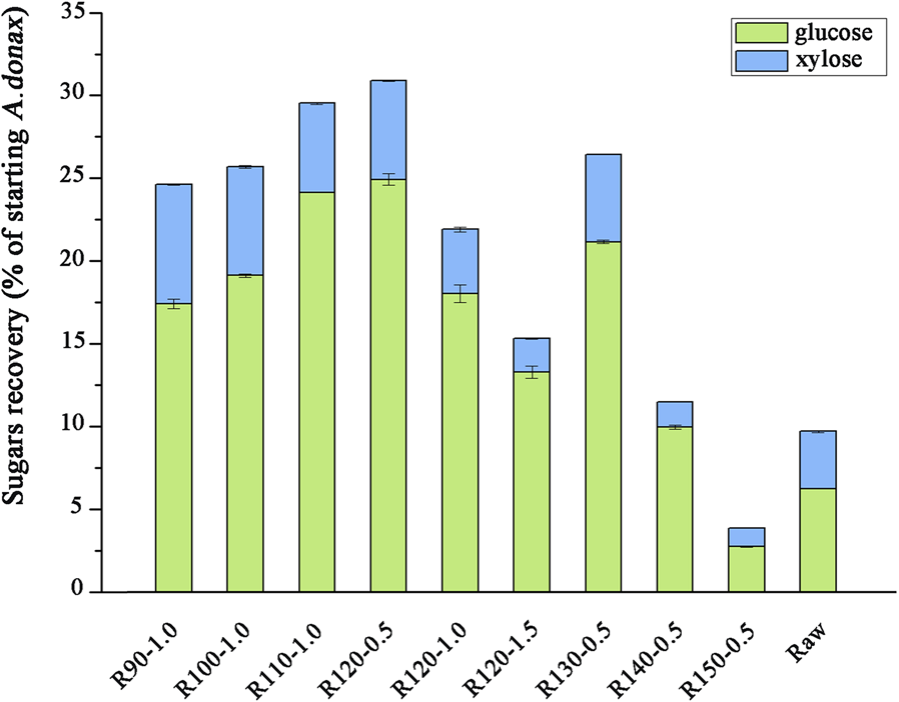
Comparison of total glucose and xylose recovery of raw and pretreated *A. donax*. after enzymatic saccharification. The sugars were recovered after 72 h of enzymatic hydrolysis at 48 °C and 150 rpm and expressed as percentage of the original biomass. *Error bars* indicate standard deviation of triplicate determinations. Sample code with definition is in Table 1

All of these pretreated *A. donax* showed a rapid response to enzyme addition, as seen in Fig. 1. As expected, the glucose released during enzymatic hydrolysis rose rapidly within the first 24 h, with a slowly released afterwards. Nevertheless, as can be seen from Table 3 that the initial rates (defined as sugar released within the first 3 h) of glucose released for samples pretreated at lower temperatures were between 60 and 92 g/kg/h compared to 92–226 g/kg/h for samples pretreated at higher temperatures. These initial rates were apparently higher than that of switchgrass subjected to ammonia fibre expansion pretreatment conducted by Bals and coworkers [36]. In addition, a number of detectable xylose was released during the saccharification for these samples (Additional file 2). Aside from the comparatively high yields of glucose and xylose, the high mass recovery at lower pretreatment temperatures resulted in higher monomeric sugars released in pretreated samples (Table 1, Fig. 2). Both R110-1.0 and R120-0.5 samples have significantly increased total sugars recovery (29.5 and 30.9 g total sugar per 100 g starting biomass) in the enzymatic hydrolysates as compared to the 8.3 and 8.4 g total sugars per 100 g starting biomass from the corresponding water pretreated samples (Table 3, Additional file 1), which was higher than those of Arabidopsis pretreated with [C_2_mim][OAc] at 140 °C for 3 h [37].

**Table 3:** Enzymatic saccharification and products in pretreatment liquor of raw and pretreated *A. donax*

Samples	Enzymatic hydrolysis			Pretreatment liquor^a^						
	Initial enzymatic rate (g/kg/h)^b^	Reducing sugar (g/100 g)^c^	Cellulose digestibility (%)^d^	TRS (%)	ASL (%)	Lactic acid (%)	Acetic acid (%)	Formic acid (%)	HMF (%)	FF (%)
Raw	17	9.7	12.5	–	–	–	–	–	–	–
R90-1.0	87	24.6	36.7	2.9	0.8	4.98	ND^e^	ND	ND	ND
R100-1.0	57	25.7	41.9	5.1	1.4	4.90	ND	ND	ND	ND
R110-1.0	92	29.6	66.7	28.8	2.6	4.70	ND	ND	ND	ND
R120-0.5	146	30.9	66.7	18.1	2.4	4.96	ND	ND	ND	ND
R120-1.0	167	21.9	73.2	31.8	3.7	4.96	ND	ND	ND	ND
R120-1.5	226	15.3	90.2	43.0	5.8	5.94	ND	ND	ND	ND
R130-0.5	149	26.4	65.2	23.5	3.3	5.06	ND	ND	ND	ND
R140-0.5	177	11.5	69.6	40.4	5.8	5.72	ND	ND	ND	ND
R150-0.5	92	3.9	41.0	35.2	6.1	5.56	ND	ND	ND	ND

All data are means of three replicates

^a^Data referred to the products in pretreatment liquor are based on dry raw materials

^b^Sugar released within the first 3 h based on dry pretreated sample

^c^Sum of glucose and xylose released in the enzymatic hydrolysates based on dry raw materials

^d^Data are calculated after 72 h of enzymatic hydrolysis at 48 °C

^e^No detectable levels (below 0.1 % based on dry raw materials)

### Structural analysis in the pretreated *Arundo donax*

As is well known, the cellulose digestibility is closely connected to the physicochemical characteristics of biomass. Morphological, chemical, and structural changes of biomass were occurred during the pretreatments to facilitate the biomass more susceptible to cellulase. Detailed analysis was conducted to compare the inherent features of the raw and differently pretreated substrates.

Modification of the gross morphology of the *A. donax* samples was analyzed by scanning electron microscopy (SEM) (Additional file 3). Results show that the raw *A. donax* had a densified smooth surface and intact fibers arranged in bundles, which prevented the accessibility of cellulase to substrates. The isothermal IL-Amberlyst pretreatment variables resulted in considerable damage in structure, exposing more cellulosic materials and contributed to high cellulose digestibility. The images of samples pretreated at low temperatures showed reversible swelling of cellulose with evidence of the twisting of separated fibres and rough surface with some cracks and fragmentations appearing when the pretreatment temperatures increased to 110 °C (Additional files 3, 4). A possible reason was the incipient disruption of hemicellulose and the onset of the delignification during the pretreatment. Re-deposition of polymers on the surface of fibre was observed in samples pretreated at high temperatures similar to those seen on wheat straw pretreated by thermal autoclaving and alkaline peroxide pretreatments [38]. When prolonging the acid reaction time, rougher and porous surfaces were visualized due to re-deposition of polymers, hemicellulose solubilisation, and lignin delignification. The increasing roughness and porosity improved the accessibility of the enzyme to fibres as reported in the previous literature [35]. Under harsher conditions (150 °C, 0.5 h), the SEM image shows disaggregation of cell bundles and the formation of sheet-like structure with large amount of re-deposited polymers on the surface of fibres (Additional file 3). These polymers were insoluble in acid solution and added to acid insoluble lignin in R150-0.5 sample. The resulted high insoluble polymers in R150-0.5 sample evidently impeded the effective enzymatic hydrolysis (Fig. 1).

Fourier transform infrared spectroscopy (FTIR) was applied to probe the chemical differences among the raw and pretreated *A. donax* (Additional file 5). When compared with the raw materials, the absorption bands at 1735 and 1240 cm^−1^ which are assigned to C=O stretching vibration in acetyl groups and C–O stretching of hemicelluloses [39], respectively, were broaden in the isothermal pretreated samples. The decline of the intensities of these two bands indicated the partial removal of hemicellulose. It is clearly discernible that the intensities of bands at 1600, 1510, 1460, and 834 cm^−1^ that associated with lignin [40] increase especially in samples of R120-1.5, R140-0.5, and R150-0.5 samples, which are consistent with the increased relative contents of lignin (Table 2). Moreover, characteristic assignments of cellulose at 1635, 1424, 1155, 1038, and 896 cm^−1^ were observed.

The changes in the crystallinity of cellulose in the untreated and pretreated materials could be established by FT-IR analysis [41, 42]. The band at 1424 cm^−1^ is considered as typical of cellulose I, whereas the absorbance at 896 cm^−1^ represents cellulose II and the amorphous regions [43]. The absorbance ratio (lateral order index, LOI) of these two bands reflects the cellulose I fraction in cellulose structures. The increased intensity in band at 896 cm^−1^ in the pretreated samples (except that of R150-0.5 sample) with decreased LOI values ranging from 0.56 to 1.15 (Table 1) indicated that the cellulose I was transformed to cellulose II to some extent. Interestingly, R150-0.5 sample had LOI value as high as 13.78. It suggested that cellulose I structure may be found in cellulose of R150-0.5 sample and the amorphous regions could almost be broken under the given condition.

**Fig. 3:**
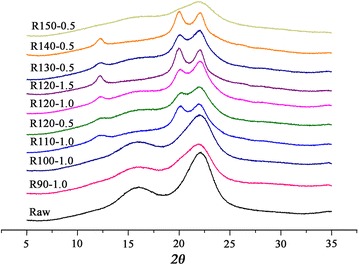
X-ray diffractograms of the raw and pretreated *A. donax*. Sample code with definition is in Table 1

X-ray diffraction (XRD) analyses were employed to further monitor the cellulose crystalline structural changes upon the pretreatment conditions varied as a whole. The crystallinity index (CrI) values were calculated (Table 1), and their XRD patterns are shown in Fig. 3. As presented, the CrI value of raw *A. donax* was 36.0 %, containing large proportions of crystalline cellulose. For pretreated samples, the CrI values were decreased to 16.5–31.4 %. After pretreatments at low temperatures, the decreased CrI values with increasing pretreatment temperatures were caused by the progressively increased swelling of cellulose in samples by IL [44]. The CrI values of samples pretreated at 120 °C successively slightly increased with increasing acid reaction time, which could be originated from the progressive degradation of amorphous hemicellulose and hydrolysis of amorphous cellulose. Further increase in the pretreatment temperature to 150 °C, highly amorphous with a minor crystalline component (CrI = 16.5 %) were noted which primary ascribed to further depolymerization of crystalline cellulose. According to Morais and coworkers, a perfect linear relationship between the CrI values and cellulose contents was found for wheat straw pretreated by imidazole [42]. However, no obvious relationship existed between cellulose content and crystallinity (both CrI and LOI values) in the present study, which may owe to the synergistic effects of ionic liquid and solid acid on *A. donax*.

The regeneration processes of IL based pretreatments can change the cellulose crystal structures by disrupting inter- and intra-chain hydrogen bonding of the cellulose fibrils in the native state. The diffraction patterns of the raw biomass exhibited typical diffractions of cellulose Ι and only the decreasing in magnitudes were detected in samples pretreated at 90–100 °C. When the samples pretreated at temperatures higher than 100 °C (excepted that of R150-0.5 sample), the peak at 22.6° shifted to lower angles and the composite peak at around 15.5° became vanishingly small. Moreover, a small shoulder peak at around 12.5° (reflection 1 1 0, cellulose II) was also observed (Fig. 3), indicating cellulose I lattice was severely distorted and partially transferred to cellulose II in these samples [44]. These results supported the observation made by FTIR analysis. It is noteworthy that samples pretreated at 120 °C, 1.5 h and 140 °C, 0.5 h displayed striking diffractions of cellulose II at 12.5°, 20.5°, and 22.1° (Fig. 3). According to Chundawat and coworkers [45] and Wada and coworkers [46], the cellulose crystal structure transformation from cellulose I to cellulose II or III_I_ increases its enzymatic hydrolysis by decreasing the number of cellulose intra-sheet hydrogen bonds. The conversion of cellulose I to expended cellulose I and II definitely increased the cellulose digestibility (Fig. 1).

### Evaluation of total reducing sugars and inhibitors in pretreatment liquor

**Fig. 4:**
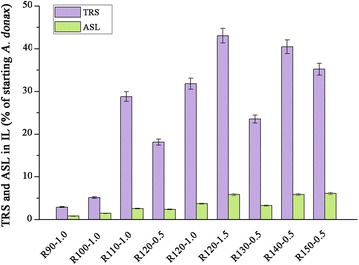
Effect of isothermal pretreatment conditions on TRS and ASL released. TRS in pretreatment liquor was measured by the DNS method. ASL in pretreatment liquor was detected at 320 nm on a UV2300 spectroscopy. The yields expressed as percentage of the original biomass. *Error bars* indicate standard deviation of triplicate determinations. Sample code with definition is in Table 1

Total reducing sugars (TRS) released during the pretreatments were determined in the IL liquors and the results are present in Fig. 4. The percentage of TRS in the IL-water phase was found to rely on the pretreatment conditions. At temperatures less than or equal to 100 °C and acid catalyzed for 1 h, only about 5 % of TRS was observed based on the starting *A. donax*, primary resulting from hemicellulose degradation. Comparatively, approximately 30 % of TRS was obtained when at an elevated temperature (110 °C). It is confirmed that the high temperature shortened the incubation period of solid acid reaction [25]. It is worth noted that there may be a strong positive correlation (*R*^2^ = 0.99) between the acid reaction time and TRS released when the pretreatment temperature is 120 °C (Additional file 6). The highest TRS yield of 43.0 % based on starting materials was found in the IL liquor of acid catalyzed for 1.5 h. This value was comparable to that obtained in the hydrolysate by pretreated the switchgrass with [C_4_mim]Cl at 160 °C for 1.5 h, followed by acidolysis by 4 M HCl at 105 °C for 2.5 h [17] and pretreated the bermuda grass with dilute acid at 121 °C for 1 h [47]. Despite similar sugars yields could be achieved by other IL based and traditional dilute acid pretreatments, this process provides a convenient, mild, and recyclable alternative method for in situ sugars production. However, a slight decrease of TRS yield in R150-0.5 sample was probably due to the decomposition of TRS to form insoluble substrates at high temperature, such as humins.

It is known that cellulose dissolved in [C_4_mim]Cl releases reducing sugars in the presence of solid acid catalysts. Our results corroborate those of other studies, such as Rinaldi and coworkers [16], Zhang and coworkers [48], Yamaguchi and coworkers [49], and Guo and coworkers [50]. They also reported that cellulose can be hydrolysed by solid acid catalysts, such as a carbon material bearing SO_3_H (100 °C, 3 h), zeolites (100 °C, 6 h) and magnetic solid acid (150 °C, 2 h). A comparatively high TRS released was detected in the IL liquors of R120-1.0, R120-1.5, R140-0.5, and R150-0.5 samples (31.8–43.0 %). Taking into account the low cellulose content (Table 2), it was demonstrated that cellulose depolymerization was the determining factor of TRS released at temperatures higher than 120 °C. It is not surprising that the relative contents of lignin increase in samples pretreated at high temperature due to the cellulose hydrolysis (Table 2). Sun and coworkers [17] have reported that sugars in IL can be extracted by a biphasic system. The recovery of sugars in [C_4_mim]Cl provides higher combined monomeric sugars released from the starting *A. donax* (Table 3).

Acid pretreatment is recognized to produce by-products considered inhibitory to microbial fermentation from carbohydrates [51, 52]. However, a quite different by-product distribution was obtained by Brønsted acid pretreatment in the present study. As can be seen from Table 3, no detectable levels (below 0.1 % based on dry raw materials) of routine inhibitors were found in any of the hydrolysates; whereas 4.7–5.94 % of lactic acid was obtained based on the dry raw materials. The lactic acid concentration obtained here was higher than that produced after 3 months of incubation by lactic acid bacteria during the solid state fermentation of forage rice round bales [53]. It was reported that cellulose can be converted to lactic acid in the presence of solid Lewis acids in water or solid Brønsted acid [20, 22]. Despite relatively high amount of lactic acid was produced during the pretreatment; this output had little effect on the further processes [52]. Lignin can also act as an inhibitor for fermentation. In terms of acid soluble lignin (ASL), 0.78–6.10 % of starting material was detected in pretreatment liquors and a similar tendency of lignin solubilizing to TRS released was seen (Table 3; Fig. 4). Regeneration of these soluble lignins from liquors is believed to reduce the inhibitory. The recovery of lactic acid and acid soluble lignin during IL-Amberlyst pretreatment may offset the high cost of IL.

### Process mass balance

**Fig. 5:**
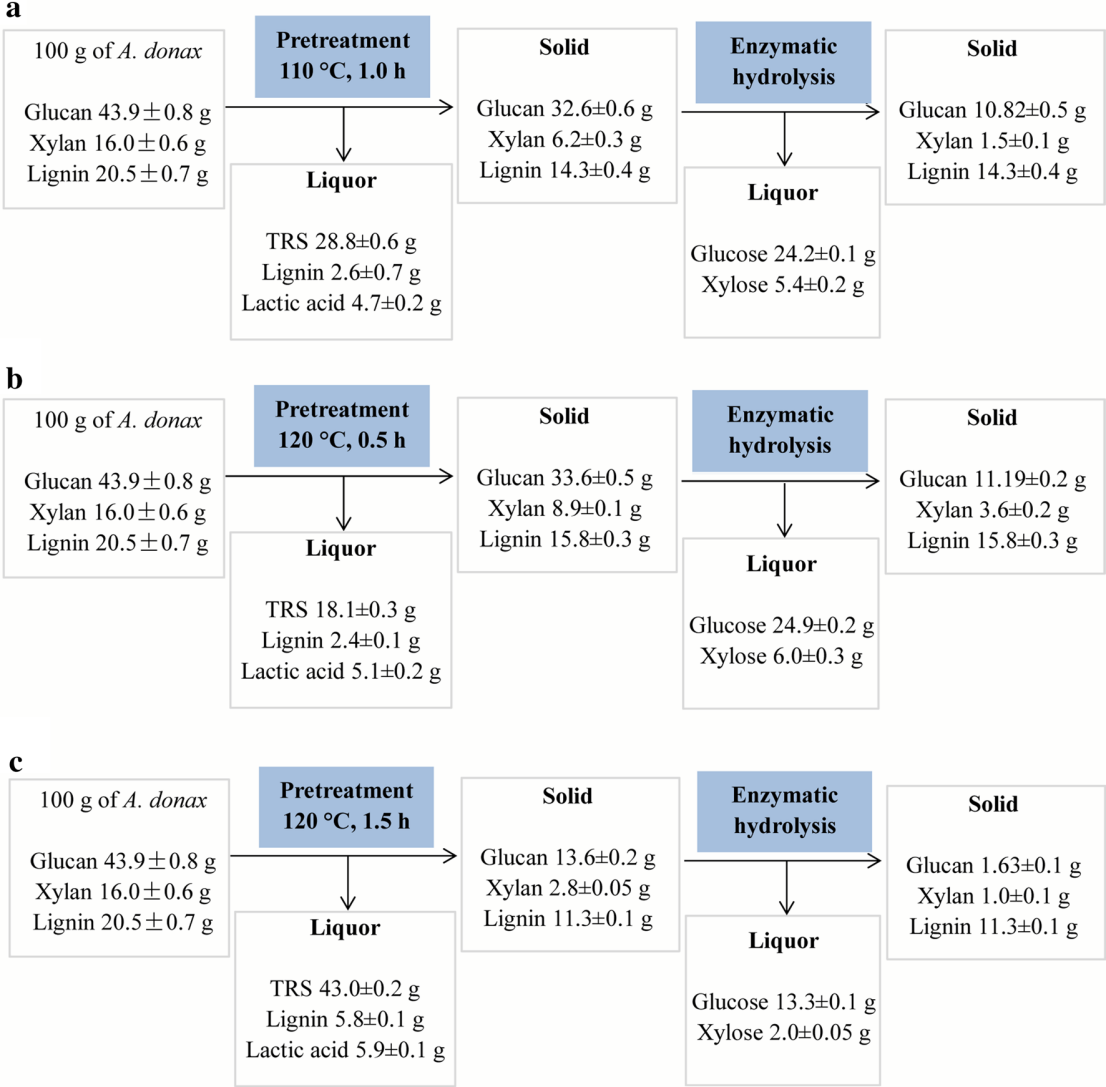
Mass balance of [C_4_mim]Cl-Amberlyst 35DRY isothermal pretreatment for the selected conditions. **a** IL pretreated at 110 °C, following by solid acid Amberlyst 35DRY catalyzed for 1.0 h, **b** IL pretreated at 120 °C, following by solid acid Amberlyst 35DRY catalyzed for 0.5 h, and **c** IL pretreated at 120 °C, following by solid acid Amberlyst 35DRY catalyzed for 1.5 h. Mass balanced adjusted to 100 g starting biomass. Values presented ±SD

In this study, the maximum TRS yield and enzymatic saccharification were achieved at 120 °C and acid catalyzed for 1.5 h, whereas the comparatively high total glucose and xylose yields in enzymatic hydrolysates were obtained at 120 °C and acid catalyzed for 0.5 h and 110 °C and acid catalyzed for 1.0 h. Therefore, process mass balances of the isothermal pretreatments were developed for these three conditions (Fig. 5). All data were based on experimental results starting from 100 g of raw *A. donax*. The pretreatments used 1900 g of IL and 20 g of Amberlyst 35DRY per 100 g of the materials. About 96 % of IL can be recycled by an aqueous biphasic system, following distillation of residual water for further reuse [54], and the solid acid resins were recovered by sieving the solid fractions.

Taking into account the cellulose conversion and mass recovery, maximum glucose and xylose produced in enzymatic hydrolysates were 24.9 and 6.0 g when biomass pretreated at 120 °C and acid catalyzed for 0.5 h. A similar glucose (24.2 g) and xylose (5.4 g) production was found at 110 °C and acid catalyzed for 1.0 h. As the TRS released increased with increasing acid reaction time, a higher combined sugars yield was obtained for the latter one. In the case of pretreatment at 110 °C and acid catalyzed for 1.0 h, 85.0 % of total reducing glycan (glucan, xylan, arabinan, and galactan) in raw materials was obtained, higher than 76 % of recovery from corn stover subjected to dilute acid pretreatment carried by Uppugundla and coworkers [29]. 4.7 g of lactic acid and 2.6 g of acid soluble lignin were produced during this pretreatment. Despite highest cellulose conversion for R120-1.5 (Fig. 1), only 32 g biomass was recovered, resulting in the low total glucose and xylose yields in enzymatic hydrolysates. However, prolonged acid reaction time enhanced the sugar released during the pretreatment. It was found that 43.0 g of TRS could be obtained in this way. Considering the enzymatic hydrolysis, it was possible to convert 84.9 % of initial total reducing glycan. Reduction of the costly hydrolytic enzymes for sugar released suggests that this process may offer compelling economic advantages. Addition to the sugars recovery, 5.9 g of lactic acid and 5.8 g of soluble lignin byproducts were also obtained which will further reduce the cost of pretreatment. Therefore, this process provides an alternative way to produce fermentable sugars cost-effectively.

## Conclusions

In this work, energy favorable facile isothermal [C_4_mim]Cl-Amberlyst 35DRY pretreatment was proposed to enhance the combined sugars yields in enzymatic hydrolysates and pretreatment liquors. During the pretreatments, it was found that hemicellulose removal, cellulose hydrolysis, crystalline conversion, and polymer re-deposition were dependent on pretreatment conditions since the liberation of H_3_O^+^ into IL were temperature- and time-dependent. Under the optimal condition IL pretreated at 110 °C for 3.0 h and subsequent acid reaction for 1.0 h, 24.2 g glucose and 5.4 g xylose in enzymatic hydrolysate and 28.8 g TRS in pretreatment liquor were produced without any notable deleterious effects. This process could potentially recover 85.0 % of the total reducing glycan in the untreated *A. donax* under a moderate temperature. Moreover, the production of lactic acid and lignin by-products will especially increase the economic prospects. Clearly, the facile isothermal [C_4_mim]Cl-Amberlyst 35DRY pretreatment provides an emerging strategy for fermentable sugars production and new revenues energy-efficiently which make IL technology a serious candidate for an industrially based biorefinery process.

## Methods

### *Arundo donax* preparation

One-year-old *A. donax* was spring harvested and kindly provided by the Beijing Academy of Agriculture and Forestry Sciences, China. The foliage was removed, and the dried stems were knife-milled and passed through 0.3 mm screen. These lignocellulosic materials were then exhaustively extracted with toluene/ethanol (2:1, v/v) in a Soxhlet apparatus, oven-dried at 40 °C for 16 h, and stored in a sealed bag at room temperature for further use. The dry matter content in biomass was 97 % (w/w).

### Ionic liquid-Amberlyst pretreatment

1-butyl-3-methylimidazolium chloride, [C_4_mim]^+^Cl^−^ with stated purity ≥98.5 %, was purchased from Lanzhou Institute of Chemical Physics of the Chinese Academy of Sciences (Lanzhou, China) and used as the IL for all pretreatments. The water content in IL was 960 ppm as determined by a volumetric Karl-Fischer titration. Solid acid Amberlyst 35DRY was acquired from the Dow Chemical Company (Michigan, America) and sieved to 20 mesh (0.425–0.850 mm). The isothermal pretreatments were performed in a 250 mL dried three-neck flask. Firstly, 5.0 g of *A. donax* was pretreated with 95.0 g of [C_4_mim]Cl at 90, 100, 110, 120, 130, 140, and 150 °C with stirring at 100 rpm for 3 h, respectively. Then 1 g of Amberylst catalyst was isothermally added to the corresponding slurry and kept at 90, 100, or 110 °C for 1.0 h, 120 °C for different periods (0.5, 1.0, or 1.5 h), or 130, 140, or 150 °C for 0.5 h with stirring at 100 rpm. After reaction time was completed, 300 mL of deionized water was added whilst stirring vigorously to precipitate the biomass. The mixture of IL, water, biomass, and solid resin was centrifuged to separate the liquid (IL and water) and solid (regenerated biomass and resin) phases. The recovered solids were washed thoroughly with hot water for 4–6 times to remove residual IL and freeze-dried. The pretreated *A. donax* samples were then gained by passing through a 0.425 mm sieve and used for analysis. Water pretreatments following the same temperature and time profiles were conducted to compare the cellulose digestibility.

### Compositional analysis

The chemical composition and acetyl content of raw and pretreated *A. donax* samples were determined according to analytical procedures established by NREL [55, 56]. More details were described in the literature [26]. All assays were performed in triplicate.

### Enzymatic saccharification

The enzymatic saccharification of raw and pretreated *A. donax* samples was performed in a 0.05 M sodium acetate buffer with a pH of 4.8 at a biomass loading of 2 % (w/v) in an air-shaking incubator at 48 °C and 150 rpm for 72 h. For hydrolysis reaction, 25 mg protein/g substrate of cellulase cocktail (Novozyme) was used to reach an enzyme loading of 20 filter paper units (FPU)/g substrate. To monitor hydrolysis kinetics, 100 μL of hydrolyzed slurry was taken periodically (0, 0.5, 3, 6, 12, 24, 48, and 72 h). The hydrolysis reaction was terminated by boiling the hydrolyzed slurry for 5 min. After cooling, the hydrolyzed slurry was centrifuged at 10,000*g* for 5 min and then the supernatant was collected and filtered through a 0.45 μm syringe filter for HPAEC analysis. Results are presented as percentage of the corresponding theoretical glucose yield of each sample. All assays were performed in triplicate.

### Fourier transform infrared spectroscopy

FTIR spectra of samples were obtained with a Thermo Scientific Nicolet iN 10 FTIR Microscopy (Madison, America) equipped with a liquid nitrogen cooled MCT detector. Spectra were collected from 1800 to 800 cm^−1^ at room temperature. 32 scans were taken for each sample and the distinguishability was 4 cm^−1^. The FTIR absorption spectra of raw and pretreated *A. donax* were baseline correction and normalized to the highest peak [40].

### X-ray diffraction

Crystallinity of raw and pretreated *A. donax* samples was analyzed by powder X-ray diffraction in a D8 Advance instrument (Bruker AXS) employing Ni-filtered Cu Kα radiation (wavelength = 0.154 nm) at 40 kV and 30 mA. Scans were obtained from 5º to 35º 2*θ* (Bragg angle) at 0.03º per second of scanning rate and at room temperature. Sample was packed into an aluminum sample holder and run as received for data collection. Sample crystallinity, as expressed by crystallinity index (CrI) was measured from the XRD data and calculated with the following formula proposed by Segal et al. [57]:

$${\text{CrI}} = \frac{{I_{ 0 0 2} - I_{\text{am}} }}{{I_{ 0 0 2} }} \times 1 0 0$$

in which *I*_002_ is the scattered intensity of 002 peak (cellulose I) at about 2*θ* = 22.5°, and *I*_am_ is the scattered intensity of peak in the amorphous phase at about 2*θ* = 18.5°.

### Scanning electron microscopy

The morphology of raw and pretreated *A. donax* samples was examined by scanning electron microscopy. The SEM images were recorded with a Hitachi S-3400 N scanning electron microscope operated at 10 kV acceleration voltages. All the samples were spread on a metal-cylinder plate with a carbon tape and coated with gold prior to examination.

### Analysis of pretreatment liquors

Total reducing sugars in ionic liquid solution were colorimetrically measured at 540 nm on a UV spectrophotometer using the DNS method [58]. D-glucose was applied as a standard and the concentration of TRS was calculated based on a standard curve obtained with D-glucose (A = 0.1404c−0.0888, R^2^ = 0.9994). For acid soluble lignin determination, the ionic liquid solution was diluted to bring the absorbance at 320 nm into the range of 0.7–1.0. Inhibitors (lactic acid, acetic acid, HMF, formic acid, and furfural) in ionic liquid solution were analyzed using an Agilent 1200 high performance liquid chromatography (HPLC) instrument equipped with a Bio-Rad HPX-87P ion-exclusion column. Degassed 0.01 N dilute H_2_SO_4_ solution was used as the mobile phase with a flow rate of 0.6 ml/min, while the temperature in the column was kept constant at 50 °C. All assays were carried out in triplicate.

## Additional files

10.1186/s13068-016-0589-8 Enzymatic hydrolysis of raw and water pretreated *A. donax*. The raw samples were exposed to the same temperature profiles as that of IL-Amberlyst pretreatments. Data are means of three replicates. Ratio = *digestibility*_*IL*-*Amberlyst*_/*digestibility*_*hot water*_.
10.1186/s13068-016-0589-8 Xylose released during enzymatic hydrolysis. Error bars indicate standard deviation of triplicate determinations. Sample code with definition is in Table 1.
10.1186/s13068-016-0589-8 SEM images of the raw and [C_4_mim]Cl-Amberlyst 35DRY isothermal pretreated *A. donax*. at magnification ×2000. Sample code with definition is in Table 1.
10.1186/s13068-016-0589-8 SEM images of the raw and [C_4_mim]Cl-Amberlyst 35DRY isothermal pretreated *A. donax*. at magnification ×100. Sample code with definition is in Table 1.
10.1186/s13068-016-0589-8 FTIR spectra of the raw and [C_4_mim]Cl-Amberlyst 35DRY isothermal pretreated *A. donax*. The band at 896 cm^−1^ is attributed to glucose ring stretch, C1-H deformation, which represents cellulose II and the amorphous regions. The band at 1043 cm^−1^ is assigned to C–O stretching vibration in cellulose and hemicelluloses. The shift of this band to lower wavenumber supported a significant loss of crystallinity of cellulose after treatment. The band at 1424 cm^−1^ referred to CH2 scissoring at C (6) in cellulose, which is considered as typical of cellulose I. The dashed line on the left referred to band at 1424 cm^−1^, the middle one referred to band at around 1043 cm^−1^, and the right one referred to the band at 896 cm^−1^. Sample code with definition is in Table 1.
10.1186/s13068-016-0589-8 Experimental data and the simulated line for TRS released as a function of acid reaction time. The pretreatment temperature was 120 °C. Data were means of three replicates. A positive correlation between the acid reaction time and TRS released was found: TRS _yield_ = 24.9415 × t + 6.02925 (*R* ^2^ = 0.99).

## Abbreviations

IL: ionic liquid
[C_4_mim]Cl: 1-*n*-butyl-3-methylimidazolium chloride
[C_2_mim][OAc]: 1-ethyl-3-methylimidazolium acetate
TRS: total reducing sugars
AIL: acid insoluble lignin
ASL: acid soluble lignin
FF: furfural
HMF: 5-hydroxymethyl furfural
SEM: scanning electron microscopy
FTIR: Fourier transform infrared spectroscopy
XRD: X-ray diffraction
CrI: crystallinity index
