# Supplementary material for: Facile isothermal solid acid catalyzed ionic liquid pretreatments to enhance the combined sugars production from Arundo donax Linn
Source: Biotechnol Biofuels. 2016 Aug 24;9(1):177. doi: 10.1186/s13068-016-0589-8 (PMC4995755; doi:10.1186/s13068-016-0589-8)
Supplement: Supplementary file 1 — 10.1186/s13068-016-0589-8 Enzymatic hydrolysis of raw and water pretreated A. donax. The raw samples were exposed to the same temperature profiles as that of IL-Amberlyst pretreatments. Data are means of three replicates. Ratio = digestibility IL-Amberlyst/digestibility hot water. [file 13068_2016_589_MOESM1_ESM.docx]

**Additional file 1. Enzymatic hydrolysis of raw and water pretreated *A. donax* ^a^.**

| Conditions | Cellulose digestibility (%) | Sugars recovery (g/100 g raw materials) | Ratio ^b^ |
| --- | --- | --- | --- |
| 90 °C 4 h | 13.6 | 7.0 | 2.7 |
| 100 °C 4 h | 13.7 | 8.1 | 3.1 |
| 110 °C 4 h | 15.0 | 8.3 | 4.5 |
| 120 °C 3.5 h | 12.5 | 8.2 | 5.3 |
| 120 °C 4 h | 13.1 | 9.3 | 5.6 |
| 120 °C 4.5 h | 14.4 | 9.2 | 6.3 |
| 130 °C 3.5 h | 13.0 | 10.0 | 5.0 |
| 140 °C 3.5 h | 14.6 | 9.1 | 4.8 |
| 150 °C 3.5 h | 18.2 | 12.2 | 2.3 |
| Untreated | 12.5 | 9.7 | 1.0 |

^a^ The raw samples were exposed to the same temperature profiles as that of IL-Amberlyst pretreatments. Data are means of three replicates. ^b^ Ratio=*digestibility _IL-Amberlyst_*/*digestibility _hot water_*.
