# Supplementary material for: Facile isothermal solid acid catalyzed ionic liquid pretreatments to enhance the combined sugars production from Arundo donax Linn
Source: Biotechnol Biofuels. 2016 Aug 24;9(1):177. doi: 10.1186/s13068-016-0589-8 (PMC4995755; doi:10.1186/s13068-016-0589-8)

**Additional file 2. Xylose released during enzymatic hydrolysis.** Error bars indicate standard deviation of triplicate determinations. Sample code with definition is in Table 1.


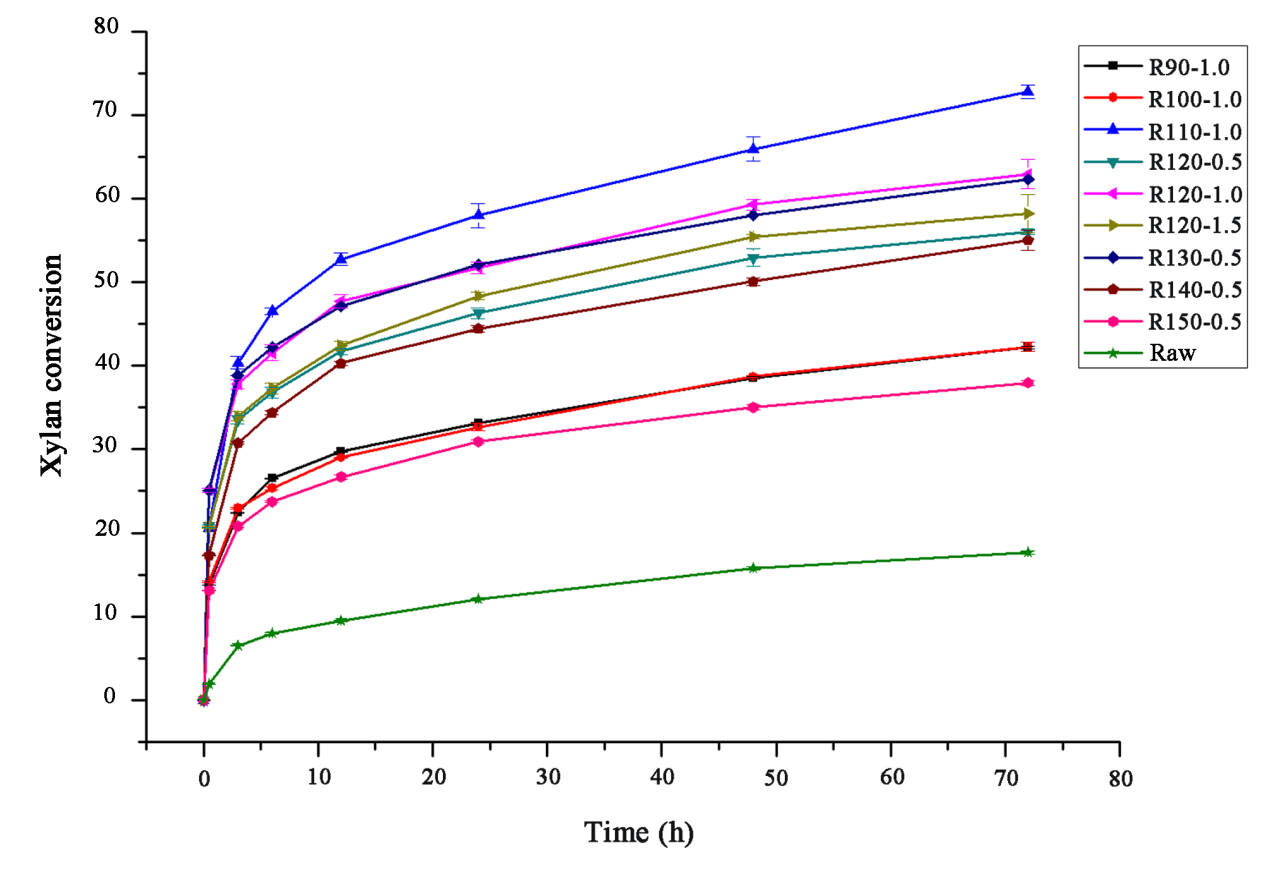

Supplement: Supplementary file 2 — 10.1186/s13068-016-0589-8 Xylose released during enzymatic hydrolysis. Error bars indicate standard deviation of triplicate determinations. Sample code with definition is in Table 1. [file 13068_2016_589_MOESM2_ESM.docx]
