# Supplementary material for: Facile isothermal solid acid catalyzed ionic liquid pretreatments to enhance the combined sugars production from Arundo donax Linn
Source: Biotechnol Biofuels. 2016 Aug 24;9(1):177. doi: 10.1186/s13068-016-0589-8 (PMC4995755; doi:10.1186/s13068-016-0589-8)

**Additional file 3.** **SEM images of the raw and [C_4_mim]Cl-Amberlyst 35DRY isothermal pretreated *A. donax*. at magnification × 2000.** Sample code with definition is in Table 1


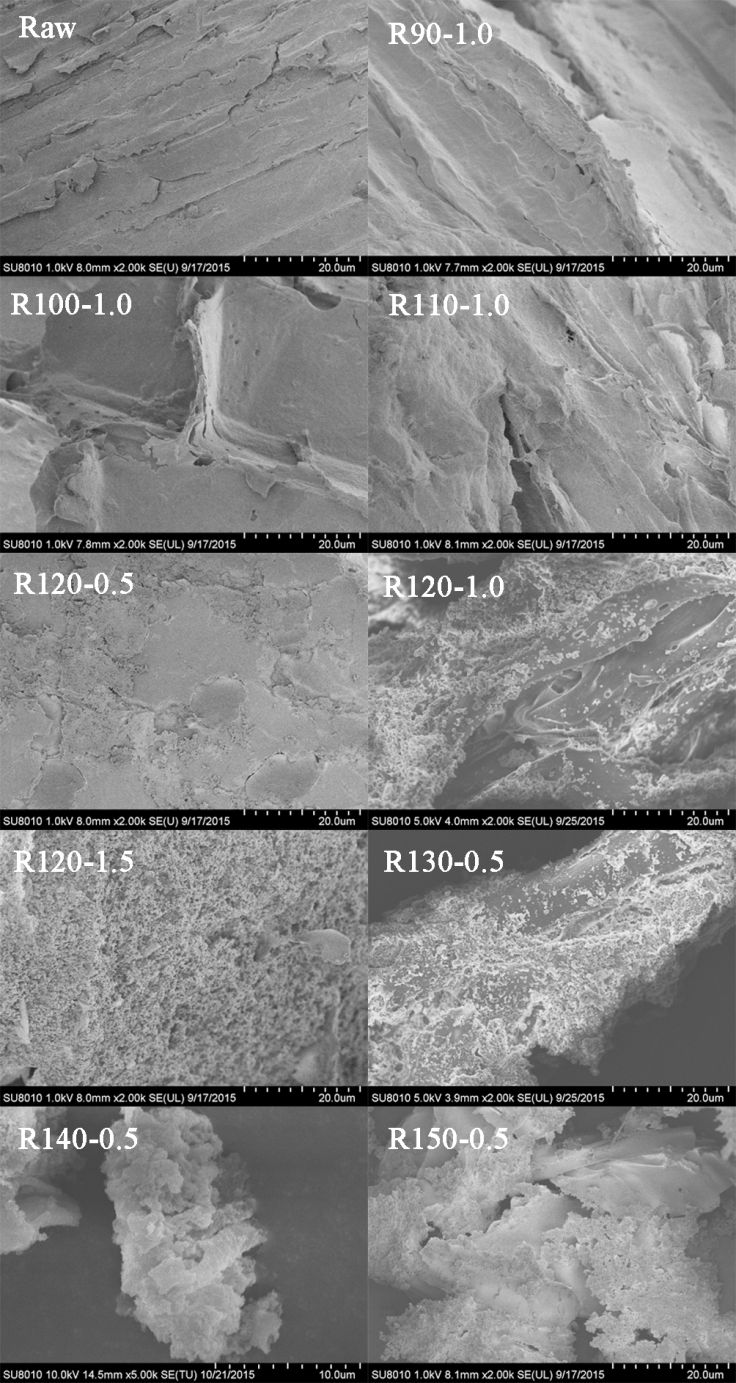

Supplement: Supplementary file 3 — 10.1186/s13068-016-0589-8 SEM images of the raw and [C4mim]Cl-Amberlyst 35DRY isothermal pretreated A. donax. at magnification ×2000. Sample code with definition is in Table 1. [file 13068_2016_589_MOESM3_ESM.docx]
