# Supplementary material for: Facile isothermal solid acid catalyzed ionic liquid pretreatments to enhance the combined sugars production from Arundo donax Linn
Source: Biotechnol Biofuels. 2016 Aug 24;9(1):177. doi: 10.1186/s13068-016-0589-8 (PMC4995755; doi:10.1186/s13068-016-0589-8)

**Additional file 5. FTIR spectra of the raw and [C_4_mim]Cl-Amberlyst 35DRY isothermal pretreated *A. donax*.** The band at 896 cm^-1^ is attributed to Glucose ring stretch, C1-H deformation, which represents the amorphous regions. The band at 1043 cm^-1^ is assigned to C-O stretching vibration in cellulose and hemicelluloses. The shift of this band to lower wavenumber supported a significant loss of crystallinity of cellulose after treatment. The band at 1424 cm^-1^ referred to CH2 scissoring at C (6) in cellulose, which is considered as typical of crystalline regions of cellulose. The dashed line on the left referred to band at 1424 cm^-1^, the middle one referred to band at around 1043 cm^-1^, and the right one referred to the band at 896 cm^-1^. Sample code with definition is in Table 1.


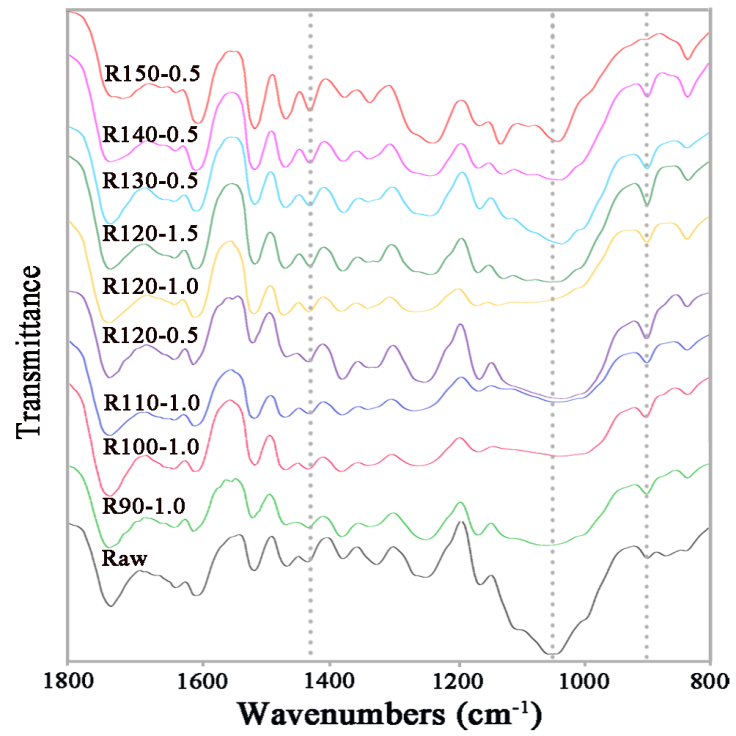

Supplement: Supplementary file 5 — 10.1186/s13068-016-0589-8 FTIR spectra of the raw and [C4mim]Cl-Amberlyst 35DRY isothermal pretreated A. donax. The band at 896 cm−1 is attributed to glucose ring stretch, C1-H deformation, which represents cellulose II and the amorphous regions. The band at 1043 cm−1 is assigned to C–O stretching vibration in cellulose and hemicelluloses. The shift of this band to lower wavenumber supported a significant loss of crystallinity of cellulose after treatment. The band at 1424 cm−1 referred to CH2 scissoring at C (6) in cellulose, which is considered as typical of cellulose I. The dashed line on the left referred to band at 1424 cm−1, the middle one referred to band at around 1043 cm−1, and the right one referred to the band at 896 cm−1. Sample code with definition is in Table 1. [file 13068_2016_589_MOESM5_ESM.docx]
