# Supplementary material for: Facile isothermal solid acid catalyzed ionic liquid pretreatments to enhance the combined sugars production from Arundo donax Linn
Source: Biotechnol Biofuels. 2016 Aug 24;9(1):177. doi: 10.1186/s13068-016-0589-8 (PMC4995755; doi:10.1186/s13068-016-0589-8)
Supplement: Supplementary file 6 — 10.1186/s13068-016-0589-8 Experimental data and the simulated line for TRS released as a function of acid reaction time. The pretreatment temperature was 120 °C. Data were means of three replicates. A positive correlation between the acid reaction time and TRS released was found: TRS yield = 24.9415 × t + 6.02925 (R 2 = 0.99). [file 13068_2016_589_MOESM6_ESM.docx]

**Additional file 6. Experimental data and the simulated line for TRS released as a function of acid reaction time.** The pretreatment temperature was 120 °C. Data were means of three replicates. A positive correlation between the acid reaction time and TRS released was found: TRS _yield_ =24.9415×t + 6.02925 (*R*^2^=0.99).

**
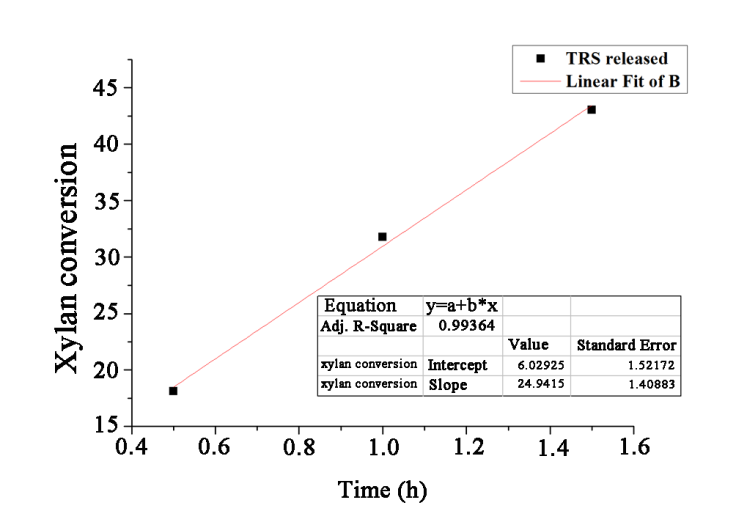
**
